# Big data and analysis of risk factors for gallbladder disease in the young generation of Korea

**DOI:** 10.1371/journal.pone.0211480

**Published:** 2019-02-22

**Authors:** Hyung Sun Kim, Seong Kyung Cho, Chang Soo Kim, Joon Seong Park

**Affiliations:** 1 Pancreatobiliary Cancer Clinic, Department of Surgery, Gangnam Severance Hospital, Yonsei University, Seoul, Korea; 2 Department of Preventive Medicine, Yonsei University College of Medicine, Seoul, Korea; McMaster University, CANADA

## Abstract

**Background/Purpose:**

Few studies have examined the risk factors for gallbladder (GB) disease in young adults. This study aimed to evaluate risk factors for GB disease in young adults based on big data in Korea.

**Methods:**

All participants underwent routine checkup at the Korea Medical Institute from June 2014 to May 2015. After excluding 677 individuals with missing information in records, 724,114 individuals (435,635 men, 288,479 women) were finally included. The definition of abnormal GB finding included stones, sludge, polyps, and adenomyomatosis detected using ultrasonography. All statistical analyses were performed using SAS software version 9.2.

**Results:**

Overall, 27,130 (17.5%) individuals were diagnosed as having abnormal GB finding in the young age group (N = 154,463, aged 20–39 years). In men, significant differences in low-density lipoprotein (LDL) and cholesterol levels were observed between the abnormal GB finding group and normal GB group (p < 0.05). In women, a significant difference in smoking history was noted between the abnormal GB finding group and normal GB group (p < 0.05). The prevalence rate of GB stones was 1.9% (27,979/154,463) in the young age group. High body mass index (BMI), large thigh circumference, and low high-density lipoprotein (HDL) level in women and low HDL level in men were independent risk factors for the presence of GB stones (p < 0.05).

**Conclusion:**

In this study, obesity-related factors (BMI, waist size, thigh circumference, and cholesterol, LDL, and HDL levels) correlated with GB disease in the young generation of Korea.

## Introduction

Gallbladder (GB) disease is a common disease of the digestive system known to occur in approximately 20% of healthy adults. In general, the prevalence of GB disease is more than double in women than in men and increases with age in both sexes, reaching approximately 30% at the age of 70 years [1–8]. In 2015, cholecystectomy ranked seventh among operative cases in Korea that were filed with the Health Insurance Review and Assessment Service [9]. Recent studies have reported a 27% growth in the number of cholecystectomies in 2015 compared with that in 2010 [9]. In Asian countries, the prevalence rate of cholelithiasis ranges from 3% to 10%; specifically, according to recent studies, the prevalence rates of cholelithiasis were 3.2% in Japan [1], 10.7% in China [2], 7.1% in North India [3], and 5.0% in Taiwan [4].

Risk factors for GB disease can be categorized into two, namely, (1) immutable factors, such as ethnicity, advanced age, female sex, and pregnancy, and (2) modifiable factors. Obesity is a major risk factor for the development of gallstones. High low-density lipoprotein (LDL), low high-density lipoprotein (HDL), and high triglyceride levels are positively correlated with gallstone formation [10, 11]. Additionally, physical activity appears to be protective, as it reduces one’s risk of developing cholelithiasis [12, 13]. Diet and lifestyle, particularly with diet westernization (increased fat), changed the stone composition from pigment to cholesterol gallstones [14–16]. The prevalence of obesity, which accounts for the largest percentage of risk factors, is increasing in Korea. Furthermore, from 1979 to 2015, the average body mass index (BMI) and waist size increased among young male and female adults [9].

Taking these states into account, we need to investigate the prevalence of GB disease in young individuals undergoing health screening owing to the change in dietary habits in recent years. Few studies have examined the risk factors for GB disease in the young population [17, 18]. Therefore, this study aimed to evaluate the risk factors for GB disease in young adults based on health checkup data in Korea.

## Materials and methods

### Study population and screening program

**Fig 1:**
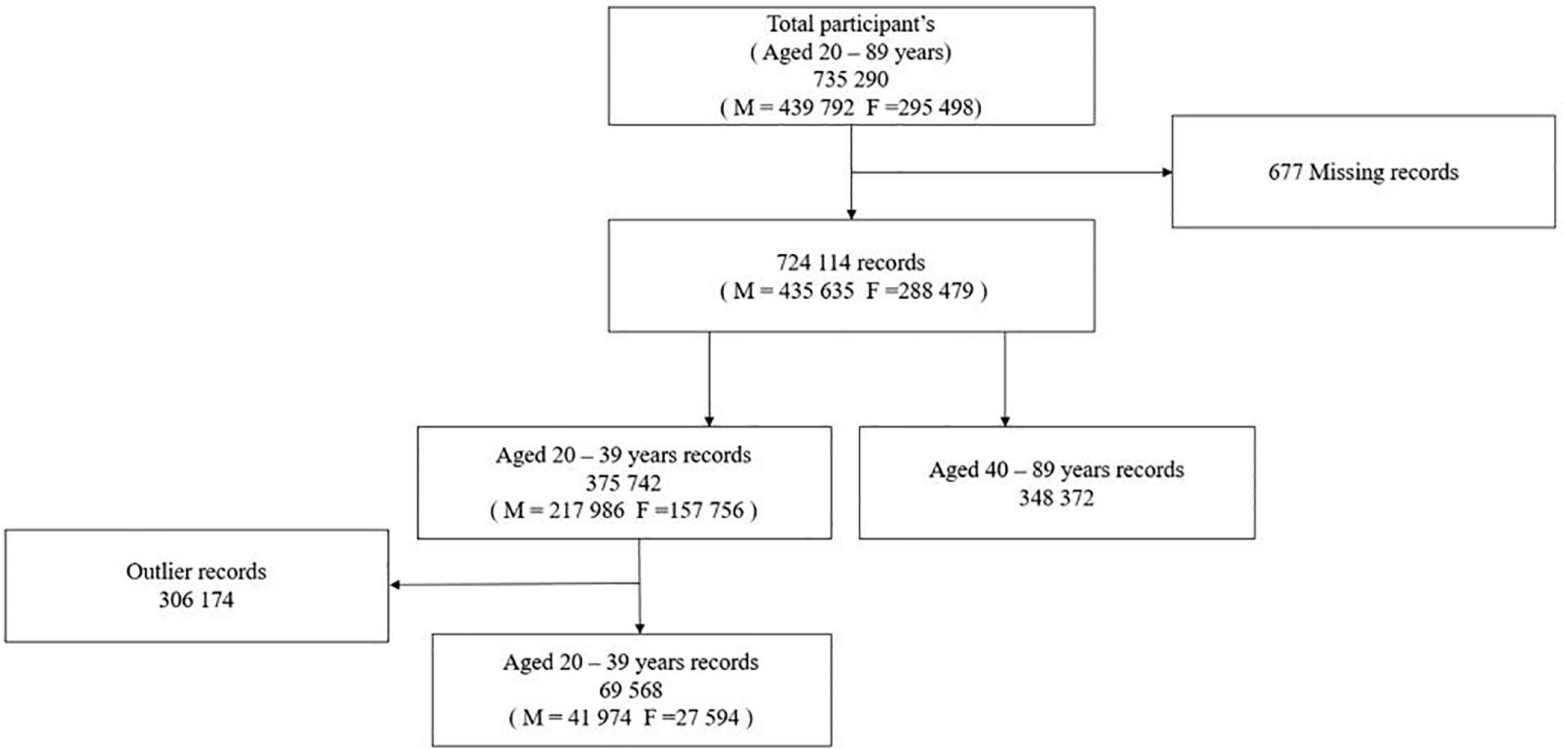
Study flow diagram.

This study was conducted using data maintained by the Korea Medical Institute (KMI) from September 2014 to August 2015. The included cohort comprised 439,792 men and 295,498 women aged 20–89 years who underwent screening via the KMI between 2014 and 2015. Among them, 677 individuals with missing information in records were excluded, and 724,114 individuals were finally included (435,635 men, 288,479 women) (Fig 1).

In this study, we reviewed young adults aged 20–39 years (217,986 male patients, 157,756 female patients) and collected data from the KMI database, which included information on participants’ anthropometric characteristics and laboratory and imaging studies. Anthropometric data included height, weight, and waist and thigh circumferences. A questionnaire that included questions about life habits (history of alcohol consumption and smoking) was used in the examination. Laboratory tests included a blood test (complete blood cell count, liver function, renal function, thyroid function, lipid profile, anemia, and tumor marker and urine analyses). Metabolic syndrome was defined as the presence of three risk factors (waist circumference ≥102 cm for men or ≥88 cm for women; triglyceride level ≥150 mg/dL; HDL level <40 mg/dL for men or <50 mg/dL for women; blood pressure ≥130/85 mmHg, and fasting glucose level ≥110 mg/dL), according to the National Cholesterol Education Program criteria [19].

The cutoff value for LDL level was 100 mg/dL in concordance with the indication by the Adult Treatment Panel III guidelines that the LDL level for initiating therapeutic lifestyle changes is ≥100 mg/dL. According to these guidelines, the optimal value for LDL level was <100 mg/dL [19]. The cutoff value for HDL level was 40 mg/dL for men and 50 mg/dL for women.

Based on average values in Koreans aged 20–30 years, the mean waist circumference is 83.08 cm for men and 73.89 cm for women, whereas the mean thigh circumference is 57.85 cm for men and 55.12 cm for women [20]. The cutoff value for BMI was 23 (BMI: overweight, 23–25 kg/m^2^; obese, 25–30 kg/m^2^; severely obese, ≥30 kg/m^2^). The cutoff value for cholesterol level was the mean value in our data. We performed further analysis of the value based on the cutoff value. Abdominal ultrasonographic findings were reported as either negative or positive (abnormal finding for the GB, liver, kidney, and pancreas). This study received approval from the institutional review board of Gangnam Severance Hospital (2015-0519-002), and informed consent was obtained from all patients.

### Case identification and data selection

The outlier group was defined based on anthropometric and laboratory data. We excluded the outlier group from the total datasets. We defined the outlier group as below 1% and above 99%. Therefore, 69,568 young (20s and 30s) men (n = 41,974) and women (n = 27,594) were included in this analysis. With respect to abnormal abdominal ultrasonographic findings, we defined abnormal GB finding as GB stones, polyps, and adenomyomatosis (Fig 1).

Data are contained within the manuscript.

### Statistical analysis

The calculated prevalence of abnormal GB finding was compared with the previously reported prevalence using tests of proportions, for which a p-value <0.05 was considered statistically significant. Anthropometric indices and laboratory values were compared for any difference, and we evaluated whether the difference was significant using paired t-test.

Continuous variables, including age, height, weight, BMI, waist and thigh circumferences, and laboratory data, are presented as mean ± standard deviation (SD) and were compared using Student’s t-test. Categorical variables, including metabolic syndrome and a history of alcohol consumption and smoking, are expressed as counts and percentages and were compared using Fisher’s exact test. The results of this model are presented as odds ratio (OR) with 95% confidence intervals (CIs) using multivariate logistic regression analysis. Data were processed using SAS software version 9.2 (SAS Institute, Cary, NC, USA).

## Results

### Baseline characteristics

Anthropometric indices and laboratory data of the young age group were analyzed (Table 1). In the young age group, the mean age of men and women was 32.9 (SD 4.2) years and 31.1 (SD 4.8) years, respectively. In addition, the mean height and weight were 172.7 (SD 19.5) cm and 74.1 (SD 12.9) kg in men and 159.9 (SD 18.8) cm and 55.4 (SD 10.2) kg in women, respectively. The BMI was 24.2 (SD 3.9) kg/m^2^ in men and 21.1 (SD 3.7) kg/m^2^ in women. A BMI of 24.2 kg/m^2^ in men is defined as overweight in Korea (BMI: overweight, 23–25 kg/m^2^; obese, 25–30 kg/m^2^; severely obese, ≥30 kg/m^2^) [10].

**Table 1:** Anthropometric indices and laboratory data of the young age group (age, 20s to 30s).

	Men (n = 41,974)	Women(n = 27,594)	P-value^a^
Age, yr	32.9±4.2	31.1±4.8	<0.01
Height, cm	172.7±19.5	159.9±18.8	<0.01
Weight, kg	74.1±12.9	55.4±10.2	<0.01
BMI	24.2±3.9	21.1±3.7	<0.01
Waist circumference, cm	81.9±11.5	69.4±10.5	<0.01
Thigh circumference, cm	53.2±7.2	49.3±7.2	<0.01
Hb [12-16g/dl]	15.5±0.8	13.1±0.8	<0.01
Hct [37–47%]	44.9±2.2	39.1±2.2	<0.01
AST [16-37U/L]	23.0±7.6	18.0±5.0	<0.01
ALT [11-46U/L]	28.0±16.8	14.2±7.9	<0.01
Bilirubin [0.3–1.8mg/dl]	0.9±0.3	0.8±0.2	<0.01
Albumin [3.4–5.3g/dl]	4.5±0.2	4.4±0.2	<0.01
r-GTP [8-46U/L]	39.8±27.8	18.1±11.4	<0.01
ALP [35-83U/L]	54.4±11.7	43.8±10.0	<0.01
Cholesterol [<200mg/dl]	190.1±30.2	177.4±26.8	<0.01
HDL [40-99mg/dl]	55.1±12.4	68.7±13.8	<0.01
LDL [<100mg/dl]	109.9±27.5	94.4±24.1	<0.01
Triglyceride [<150mg/dl]	127.1±73.2	72.1±38.8	<0.01

**Abbreviations**: yr, years; BMI, body mass index; Hb, hemoglobin; Hct, hematocrit; AST, aspartate aminotransferase; ALT, alanine aminotransferase; r-GTP, gamma-glutamyl transpeptidase; ALP, alkaline phosphatase; HDL, high-density lipoprotein; LDL, low-density lipoprotein

^a^ p values were calculated using the t test.

The mean waist and thigh circumference in the young age group were 81.9 (SD 11.5) cm and 53.2 (SD 7.2) cm in men and 69.4 (SD 10.5) cm and 49.3 (SD 7.2) cm in women, respectively. All variables showed statistically significant differences between men and women.

### Metabolic syndrome

In the young age group, 1,181 (1.7%) participants had metabolic syndrome. Among participants, 20337 (29.2%) were smokers, 69227 (99.5%) were drinkers, and 289 were diagnosed with diabetes. All variables showed statistically significant differences between men and women (Table 2).

**Table 2:** Metabolic syndrome and health behaviors in the young age group.

Variable		Men(n = 41974)	Women(n = 27594)	P -value^a^
Metabolic Syndrome	Yes	656	525	<0.01
	No	41318	27069	
Smoking	Non smoker	23664	25567	<0.01
	Smoker	18310	2027	
Drinking	Non drinker	144	197	<0.01
	Drinker	41830	27397	
Diabetes mellitus	Yes	208	81	<0.01
	No	41766	27513	

^a^ p values were calculated using the t test.

### Young age group with abnormal GB finding

#### Baseline characteristics

In men and women, there were significant differences in age values between the abnormal GB finding group and normal GB group. In men, significant differences in BMI, triglyceride level, and laboratory data (hemoglobin, hematocrit, aspartate aminotransferase, alanine aminotransferase, albumin, gamma-glutamyl transpeptidase, alkaline phosphatase) were observed between the abnormal GB finding group and normal GB group. In women, significant differences in the values of anthropometric indices (weight, BMI, waist and thigh circumferences), LDL and HDL levels, and laboratory data (hemoglobin, hematocrit, alanine aminotransferase, albumin) between the abnormal GB finding group and normal GB group (Table 3).

**Table 3:** Anthropometric indices and laboratory data of the young age group with abnormal GB finding (stones, sludge, polyps, and adenomyomatosis) and normal GB finding.

	Men (n = 7631)			Women (n = 3042)		
	Abnormal GB	Normal GB	p-value ^a^	Abnormal GB	Normal GB	p-value ^a^
Age, yr	33.5±3.9	32.9±4.2	<0.01	32.7±4.3	31.3±4.8	<0.01
Height, cm	174.8±5.4	174.7±5.6	0.41	162.0±4.9	162.0±5.1	0.85
Weight, kg	74.9±9.6	75.0±10.1	0.11	57.1±8.2	56.5±7.8	<0.01
BMI	24.5±2.8	24.6±2.9	0.03	21.7±2.9	21.5±2.8	<0.01
Waist circumference, cm	82.8±7.1	82.9±7.3	0.08	71.3±7.0	70.7±6.7	<0.01
Thigh circumference, cm	53.9±4.2	53.9±4.4	0.83	50.4±4.4	50.2±4.3	0.01
Hb [12-16g/dl]	15.4±0.8	15.5±0.8	<0.01	13.1±0.8	13.0±13.0	<0.01
Hct [37–47%]	44.8±2.2	44.9±2.2	<0.01	38.9±2.2	39.1±2.2	<0.01
AST [16-37U/L]	22.3±7.3	23.3±7.9	<0.01	17.9±5.1	18.1±5.1	0.14
ALT [11-46U/L]	26.7±15.4	28.6±17.3	<0.01	14.7±8.3	14.3±7.9	0.01
Bilirubin [0.3–1.8mg/dl]	0.9±0.3	0.9±0.3	0.56	0.8±0.2	0.8±0.3	0.15
Albumin [3.4–5.3g/dl]	4.4±0.2	4.5±0.2	<0.01	4.3±0.2	4.4±0.2	<0.01
r-GTP [8-46U/L]	39.0±27.8	40.3±28.3	<0.01	18.3±12.0	18.1±11.4	0.24
ALP [35-83U/L]	53.9±11.8	54.4±11.7	<0.01	43.6±10.4	43.7±9.9	0.72
Cholesterol [<200mg/dl]	189.6±29.9	190.7±30.3	<0.01	178.5±27.3	177.6±26.8	0.08
HDL [40-99mg/dl]	54.7±12.3	54.9±12.4	0.07	67.7±13.7	68.4±13.7	0.02
LDL [<100mg/dl]	110.3±27.1	110.3±27.5	0.94	96.3±24.3	94.7±21.2	<0.01
Triglyceride [<150mg/dl]	124.1±70.8	128.2±72.8	<0.01	72.8±39.3	72.5±38.6	0.65

**Abbreviations:** GB, gallbladder; yr, years; BMI, body mass index; Hb, hemoglobin; Hct, hematocrit; AST, aspartate aminotransferase; ALT, alanine aminotransferase; r-GTP, gamma-glutamyl transpeptidase; ALP, alkaline phosphatase; HDL, high-density lipoprotein; LDL, low-density lipoprotein

^a^ p values were calculated using the t test.

#### Risk factors for abnormal GB finding

In the univariate analysis, significant differences in cholesterol level among men were observed between the abnormal GB finding group and normal GB group (cholesterol: OR = 0.928, 95% CI = 0.882–0.976, p < 0.01). Among women, there were significant differences in waist circumference (OR = 1.177, 95% CI = 1.063–1.304, p < 0.01), thigh circumference (OR = 1.111, 95% CI = 1.021–1.20, p = 0.01), BMI (OR = 1.124, 95% CI = 1.027–1.231, p = 0.01), LDL level (OR = 1.098, 95% CI = 1.015–1.187, p = 0.02), cholesterol level (OR = 1.082, 95% CI = 0.999–1.171, p = 0.05), and smoking history (OR = 1.189, 95% CI = 1.013–1.395, p = 0.03) between the abnormal GB finding group and normal GB group (Table 4).

**Table 4:** Univariate analysis of risk factors for abnormal GB finding and GB stones.

Variables	Men				Women			
	Abnormal GB		GB stone		Abnormal GB		GB stone	
	Odds ratio	P value^b^	Odds ratio	P value^b^	Odds ratio	P value^b^	Odds ratio	P value^b^
	[95%CI]		[95%CI]		[95%CI]		[95%CI]	
Waist circumference	1.013	0.67	1.192	0.04	1.177	<0.01	2.021	<0.01
	[0.955–1.075]		[1.003–1.417]		[1.063–1.304]		[1.666–2.452]	
Thigh circumference	1.012	0.65	1.016	0.82	1.111	0.01	1.785	<0.01
	[0.961–1.066]		[0.88–1.173]		[1.021–1.209]		[1.502–2.123]	
BMI	1.004	0.88	1.204	0.01	1.124	0.01	2.079	<0.01
	[0.952–1.059]		[1.035–1.401]		[1.027–1.231]		[1.742–2.48]	
LDL	0.993	0.78	1.017	0.81	1.098	0.02	1.132	0.16
	[0.943–1.046]		[0.881–1.176]		[1.015–1.187]		[0.953–1.344]	
HDL	0.967	0.21	0.813	<0.01	0.888	0.08	0.583	<0.01
	[0.918–1.019]		[0.705–0.936]		[0.777–1.016]		[0.454–0.75]	
Cholesterol	0.928	<0.01	0.921	0.25	1.082	0.05	0.999	0.99
	[0.882–0.976]		[0.801–1.059]		[0.999–1.171]		[0.837–1.191]	
Smoking	0.989	0.65	0.921	0.25	1.189	0.03	0.854	0.32
	[0.94–1.04]		[0.801–1.06]		[1.013–1.395]		[0.625–1.167]	
Drinking	1.138	0.54	1.909	0.16	0.953	0.83	0.251	0.17
	[0.747–1.736]		[0.779–4.682]		[0.602–1.51]		[0.035–1.798]	

**Abbreviations**: GB, gallbladder; BMI, body mass index; HDL, high-density lipoprotein; LDL, low-density lipoprotein

^b^p values were calculated using the cox-proportional hazard model

**Fig 2:**
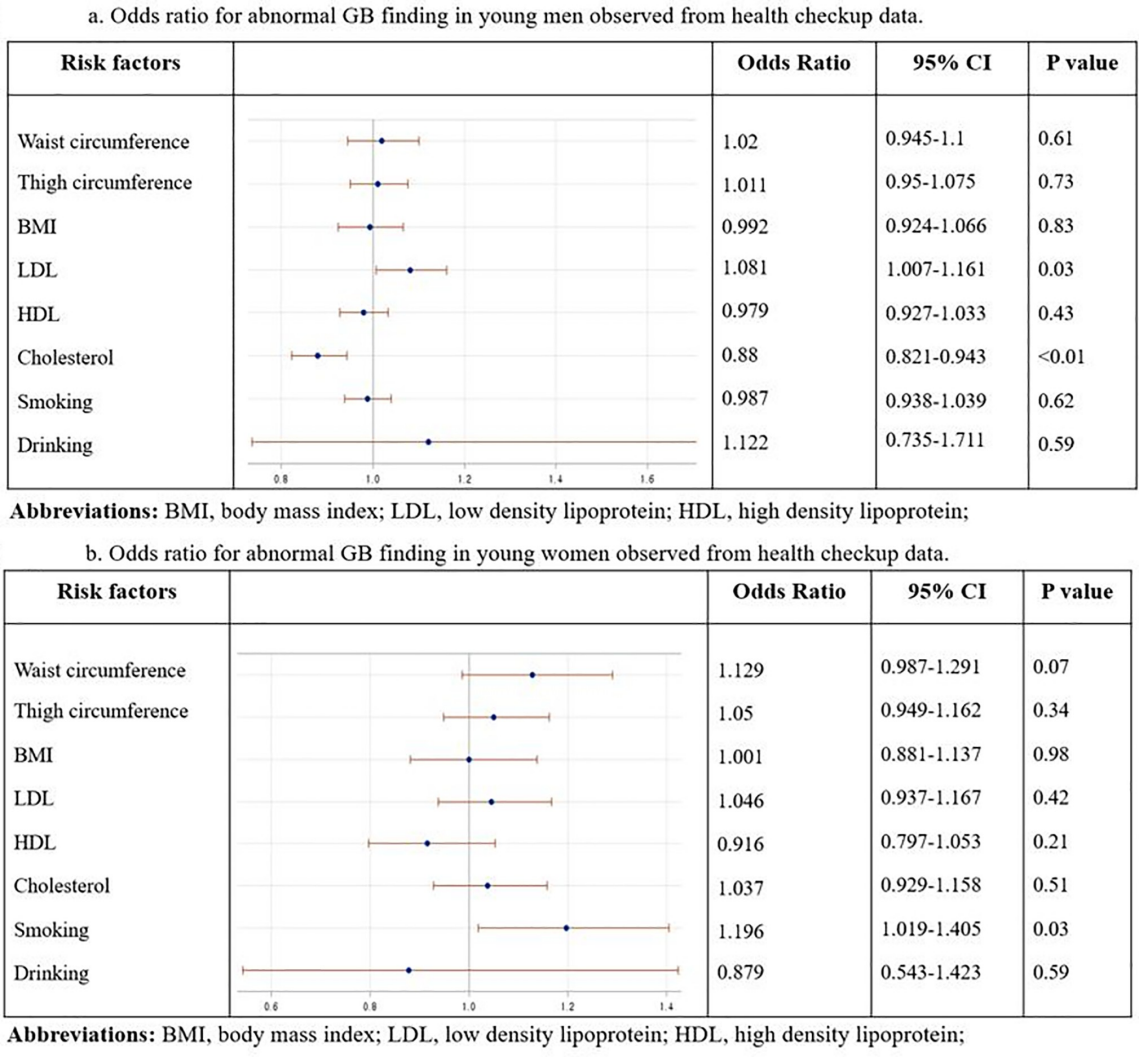
Odds ratios for abnormal GB finding in young men (A) and women (B) observed from health checkup data.

In the multivariate analysis, significant differences in LDL level (OR = 1.081, 95% CI = 1.007–1.161, p = 0.03) and cholesterol level (OR = 0.88, 95% CI = 0.821–0.943, p < 0.01) were noted among men between the abnormal GB finding group and normal GB group (Fig 2A). Furthermore, significant difference in smoking history was observed among women between the abnormal GB finding group and normal GB group (smoking history: OR = 1.196, 95% CI = 1.019–1.405, p = 0.03) (Fig 2B).

### Young age group with GB stones

We compared the GB stone group and non-GB stone group among young adults. In the univariate analysis, significant differences in waist circumference (OR = 1.192, 95% CI = 1.003–1.417, p = 0.04), BMI (OR = 1.204, 95% CI = 1.035–1.401, p = 0.01), and HDL level (OR = 0.813, 95% CI = 0.705–0.936, p < 0.01) were observed among men between the GB stone group and normal GB group. Moreover, significant differences in waist circumference (OR = 2.021, 95% CI = 1.666–2.452, p < 0.01), thigh circumference (OR = 1.785, 95% CI = 1.502–2.123, p < 0.01), BMI (OR = 2.079, 95% CI = 1.742–2.48, p < 0.01), and HDL level (OR = 0.583, 95% CI = 0.454–0.75, p < 0.01) were noted among women between the GB stone group and normal GB group (Table 4).

With respect to anthropometric indices, BMI, waist circumference, and HDL level were significant risk factors for the presence of GB stones in the male GB stone group (p < 0.01). In women, the LDL level was also a significant risk factor for the presence of GB stones (p < 0.01) (Table 5).

**Table 5:** Comparison of the GB stone group and non-GB stone group among young adults.

	Men (n = 37301)			Women (n = 22098)		
	GB stone (n = 806)	Non GB stone (n = 36495)	p-value ^a^	GB stone (n = 554)	Non GB stone (n = 21544)	p-value ^a^
BMI	24.9 ± 3.1	24.6 ± 2.9	<0.01	22.8 ± 3.6	21.5 ± 2.8	<0.01
Waist circumference, cm	83.8 ± 7.8	82.9 ± 7.3	<0.01	73.5 ± 8.2	70.7 ± 6.7	<0.01
Thigh circumference, cm	54.1 ± 4.4	53.9 ± 4.4	0.21	51.6 ± 4.9	50.2± 4.3	0.21
Cholesterol [<200mg/dl]	189.3 ± 30.9	190.5 ± 30.2	0.29	178.9 ± 28.2	177.7 ± 26.8	0.30
HDL [40-99mg/dl]	53.2 ± 11.6	55.0 ± 12.4	<0.01	65.1 ± 13.9	68.4 ± 13.7	<0.01
LDL [<100mg/dl]	110.8 ± 27.1	110.3 ± 27.5	0.61	97.9 ± 24.6	94.9 ± 24.2	<0.01

**Abbreviations**: GB, gallbladder; BMI, body mass index; HDL, high-density lipoprotein; LDL, low-density lipoprotein

^a^ p values were calculated using the t test.

**Fig 3:**
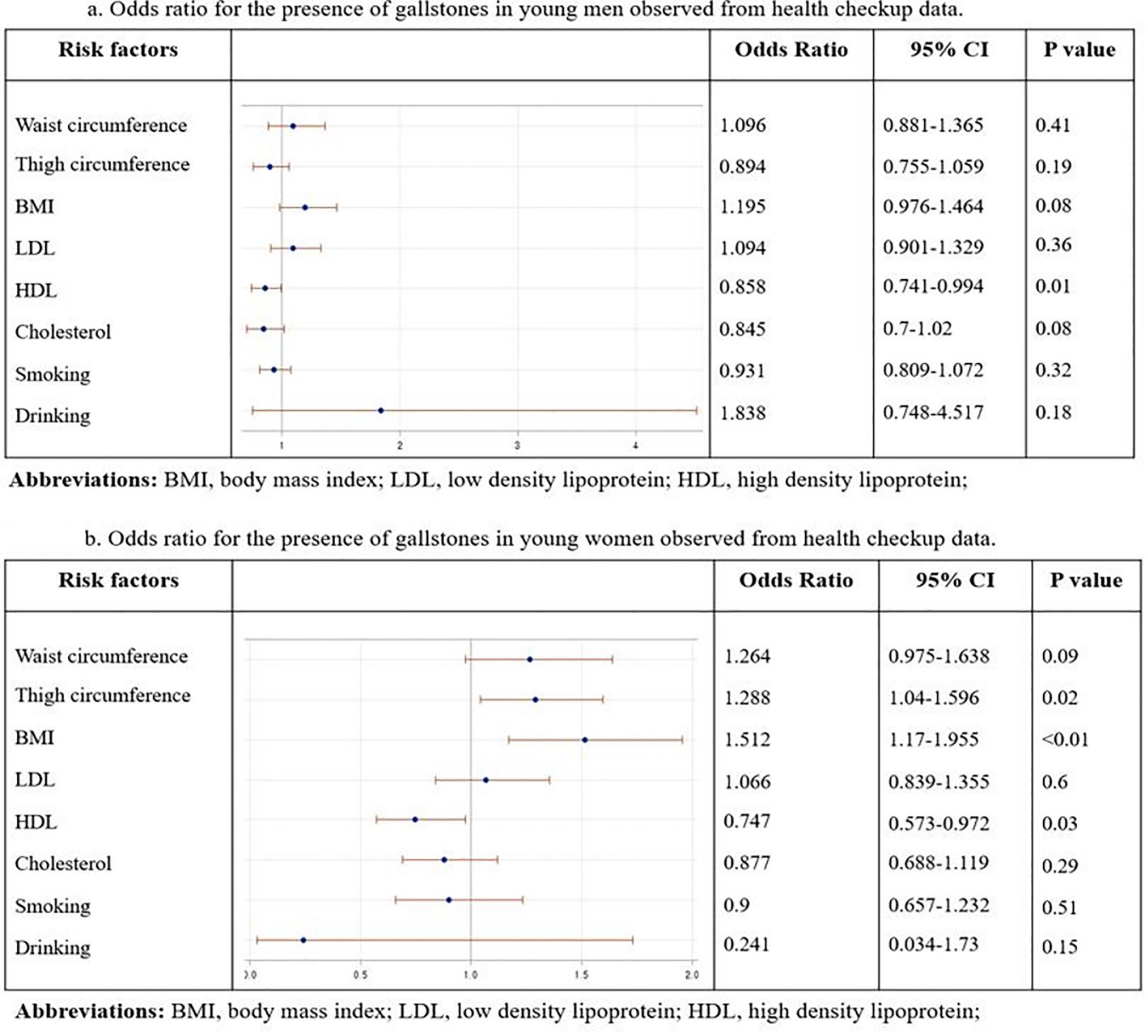
Odds ratios for the presence of gallstones in young men (A) and women (B) observed from health checkup data.

Multivariate analysis of the risk of gallstones by some variables, including anthropometric indices and obesity-related factors, was performed in the young age group. In men, low HDL level (OR = 0.858, 95% CI = 0.741–0.994, p = 0.01) was an independent risk factor for the presence of GB stones (Fig 3A). In women, high BMI (OR = 1.512, 95% CI = 1.17–1.955, p < 0.01), large thigh circumference (OR = 1.288, 95% CI = 1.04–1.596, p = 0.02), and low HDL level (OR = 0.747, 95% CI = 0.573–0.972, p = 0.03) were independent risk factors for the presence of GB stones (Fig 3B).

## Discussion

BMI, obesity, abdominal fat, metabolic syndrome, and diabetes mellitus are confirmed risk factors for gallstones [5, 10, 12, 21–24]. However, the prevalence of abnormal GB finding among young adults with a high level of obesity has not been investigated. The present study is the first large-scale study on abnormal GB findings detected by abdominal ultrasonography in healthy individuals. As the prevalence of obesity increases globally [25–27], the prevalence of gallstones associated with obesity is expected to also increase. In particular, the prevalence of obesity is rapidly increasing in Korea owing to the westernization of food and lifestyle [28]. In recent years, obesity has emerged as a major health threat in Korea [29]. The Korean National Health Insurance Service reported that the percentage of obese Koreans (those with a BMI of 30 kg/m^2^ or above) increased from 2.5% of the entire population in 2002 to 4.2% in 2012 and that the prevalence rate of GB stones in the 20s and 30s was 0.95% and 2.26%, respectively [9]. According to another study, the prevalence rate of gallstones in the early 2000s was 0.39% for those aged <30 years and 0.99% for those in their 30s [21]. In the present study, which was performed using data from September 2014 to August 2015, the prevalence rate of abnormal GB finding and GB stones was 17.5% and 1.9%, respectively. This result showed that the increase in obesity in Korea for approximately 10 years was associated with an increase in the prevalence of gallstones.

However, abdominal ultrasonography is performed to identify the cause of persistent abdominal discomfort, and several young individuals have been diagnosed with cholecystitis. Therefore, detecting young adults with asymptomatic GB disease is important because early diagnosis of GB disease can be the first step to finding the cause of indigestion and abdominal pain.

In our data, abnormal ultrasonographic findings of GB disease included cholesterol and pigment stones, polyps, and adenomyomatosis. Traditionally, pigment and cholesterol stones were thought to represent a different pathogenesis. However, recent findings have indicated that these types of stones share the same risk factors [30–32].

Variants of the gene encoding UGT1A1 (uridine 5’-diphosphate (UDP)- glucuronosyltransferase 1A1) responsible for bilirubin conjugation were correlated with the risk of gallstones and stone bilirubin content, suggesting common factors in the pathogenesis of cholesterol and pigment gallstones [31].

As these stones were not classified in our data, this was a limitation in determining the risk factors for GB disease. In this study, the mean values for all obesity-related factors among young men and women were higher in the abnormal GB finding group than in the normal GB group. This finding suggests that obesity-related factors affect GB disease in young adults.

Among men, BMI was higher in the abnormal GB finding group than in the normal GB group, which was contrary to our conclusion. BMI and waist circumference among women were higher in the abnormal GB finding group than in the normal GB group (Table 3). For this reason, metabolic factors in women seem to exert a greater effect on GB disease. Lifestyle habits, such as smoking and drinking, seem to have a greater effect on risk factors for GB disease in men.

The risk of abnormal GB finding in young men was 1.5 times higher than that in young women (OR = 1.553, 95% CI = 1.464–1.648, p < 0.01). This result is in contrast to a previous finding that indicated generally higher prevalence of GB disease in women than in men [5]. In this study, differences in the results for obesity-related factors in the abnormal GB finding group were larger in men than in women. Compared with young women, young men in Korea are familiar with the culture of having dinner and drinking with company. This culture results in more obesity, especially among young men [33, 34]. Relatively, young women prefer to have a slender appearance [35, 36], and most obesity-related factors in the abnormal GB finding group were within the normal range.

In men, obesity-related factors such as LDL level are proportional to the risk of abnormal GB finding. However, cholesterol level has the opposite effect. There was only a subtle difference in cholesterol level between the abnormal GB finding group and normal GB group; furthermore, cholesterol level was within the normal range. In women, smoking is proportional to the risk of abnormal GB finding. The role of smoking as a risk factor has not yet been clarified. Some studies have reported a correlation between smoking and GB disease [37–39]; nevertheless, the definite mechanism of smoking in GB disease remains unknown. GB disease associated with smoking may be explained by changes in serum cholesterol concentrations [38].

In this study, abdominal ultrasonography was performed in adults over 20 years of age. However, most young adults in their 20s to 40s until now do not undergo ultrasonography because abdominal ultrasonography is not covered by insurance. However, our results would be meaningful because insurance for ultrasonography is implemented in Korea starting from this year.

The present study has limitations, particularly its retrospective design and data from young, healthy workers who underwent health screening. Because of the high proportion of young individuals, the prevalence in young adults in relation to the overall prevalence is not definitely indicative of the actual prevalence. In addition, as we used data from one year in 2014, the study included recent data; nonetheless, the fact that it is not a multiyear study is a disadvantage. Additionally, the participants in the study were not followed up. Thus, we could not evaluate their treatment for GB disease (cholecystectomy or medications) after their health checkup. In our study, we did not classify each ultrasonographic finding for GB disease (cholesterol stone, pigment stone, polyp, or adenomyomatosis). We could not also evaluate each risk factor. However, for GB stones, risk factors were analyzed separately. These results were different from those of the overall analysis of GB disease. GB stones are more likely to be associated with obesity-related factors (BMI, HDL level, and thigh circumference). In this study, we could not describe the degree of alcohol consumption according to the amount of alcohol because the questionnaire used for collecting health checkup data was not designed to assess the exact amount of alcohol.

## Conclusions

This study is the first attempt to analyze the prevalence of and risk factors for GB disease in young adults based on large-scale health screening data. The use of big data aims to analyze large, heterogeneous datasets to provide in-depth insights into complex processes, and it has the advantages of evaluating risk factors. Based on these results, we might consider the probability of GB disease in young adults with digestive symptoms, such as persistent abdominal discomfort.

## Supporting information

S1 File Raw data of health checkup. (XLSX)
