# Correction: Big data and analysis of risk factors for gallbladder disease in the young generation of Korea

**DOI:** 10.1371/journal.pone.0213950

**Published:** 2019-03-13

**Authors:** Hyung Sun Kim, Seong Kyung Cho, Chang Soo Kim, Joon Seong Park

In the Results subsection of the Abstract, there is an error in the fourth sentence. The correct sentence is: The prevalence rate of GB stones was 1.9% (2,979/154,463) in the young age group.

There is an error in Table 3. Please see the correct Table 3 here.

**Table 3:** Anthropometric indices and laboratory data of the young age group with abnormal GB finding (stones, sludge, polyps, and adenomyomatosis) and normal GB finding.

	Men (n = 37301)			Women (n = 22098)		
	Abnormal GB (n = 7631)	Normal GB (n = 29670)	p-value ^a^	Abnormal GB (n = 3042)	Normal GB (n = 19056)	p-value ^a^
Age, yr	33.5±3.9	32.9±4.2	<0.01	32.7±4.3	31.3±4.8	<0.01
Height, cm	174.8±5.4	174.7±5.6	0.41	162.0±4.9	162.0±5.1	0.85
Weight, kg	74.9±9.6	75.0±10.1	0.11	57.1±8.2	56.5±7.8	<0.01
BMI	24.5±2.8	24.6±2.9	0.03	21.7±2.9	21.5±2.8	<0.01
Waist circumference, cm	82.8±7.1	82.9±7.3	0.08	71.3±7.0	70.7±6.7	<0.01
Thigh circumference, cm	53.9±4.2	53.9±4.4	0.83	50.4±4.4	50.2±4.3	0.01
Hb [12-16g/dl]	15.4±0.8	15.5±0.8	<0.01	13.1±0.8	13.0±13.0	<0.01
Hct [37–47%]	44.8±2.2	44.9±2.2	<0.01	38.9±2.2	39.1±2.2	<0.01
AST [16-37U/L]	22.3±7.3	23.3±7.9	<0.01	17.9±5.1	18.1±5.1	0.14
ALT [11-46U/L]	26.7±15.4	28.6±17.3	<0.01	14.7±8.3	14.3±7.9	0.01
Bilirubin [0.3–1.8mg/dl]	0.9±0.3	0.9±0.3	0.56	0.8±0.2	0.8±0.3	0.15
Albumin [3.4–5.3g/dl]	4.4±0.2	4.5±0.2	<0.01	4.3±0.2	4.4±0.2	<0.01
r-GTP [8-46U/L]	39.0±27.8	40.3±28.3	<0.01	18.3±12.0	18.1±11.4	0.24
ALP [35-83U/L]	53.9±11.8	54.4±11.7	<0.01	43.6±10.4	43.7±9.9	0.72
Cholesterol [<200mg/dl]	189.6±29.9	190.7±30.3	<0.01	178.5±27.3	177.6±26.8	0.08
HDL [40-99mg/dl]	54.7±12.3	54.9±12.4	0.07	67.7±13.7	68.4±13.7	0.02
LDL [<100mg/dl]	110.3±27.1	110.3±27.5	0.94	96.3±24.3	94.7±21.2	<0.01
Triglyceride [<150mg/dl]	124.1±70.8	128.2±72.8	<0.01	72.8±39.3	72.5±38.6	0.65

**Abbreviations:** GB, gallbladder; yr, years; BMI, body mass index; Hb, hemoglobin; Hct, hematocrit; AST, aspartate aminotransferase; ALT, alanine aminotransferase; r-GTP, gamma-glutamyl transpeptidase; ALP, alkaline phosphatase; HDL, high-density lipoprotein; LDL, low-density lipoprotein

^a^ p values were calculated using the t test.
